# Repeated Liver Resection for Colorectal Liver Metastases: A Comparison with Primary Liver Resections concerning Perioperative and Long-Term Outcome

**DOI:** 10.1155/2012/568214

**Published:** 2012-08-29

**Authors:** Kristoffer Jönsson, Gerd Gröndahl, Martin Salö, Bobby Tingstedt, Roland Andersson

**Affiliations:** Department of Surgery, Clinical Sciences Lund, Lund University and Skåne University Hospital Lund, Lund SE-22185, Sweden

## Abstract

*Introduction*. 60% of patients operated for colorectal liver metastases (CRLM) will develop recurrent disease and some may be candidates for a repeated liver resection. The study aimed to evaluate differences in intraoperative blood loss and complications comparing the primary and the repeated liver resection for metastases of colorectal cancer (CRC), as well as to evaluate differences in long-time follow-up. *Method*. 32 patients underwent 34 repeated liver resections due to recurrence of CRLM an studied retrospectively to identify potential differences between the primary and the repeat resections. *Results*. There was no 30-day postoperative mortality or postoperative hospital deaths. The median blood loss at repeat resection (1850 mL) was significantly (*P* = 0.014) higher as compared to the primary liver resection (1000 mL). This did not have any effect on the rate of complications, even though increased bleeding in itself was a risk factor for complications. There were no differences in survival at long-term follow-up. *Discussion*. A repeated liver resection for CRLM was associated with an increased intraoperative bleeding as compared to the first resection. Possible explanations include presence of adhesions, deranged vascular anatomy, more complicated operations and the effects on the liver by chemotherapy following the first liver resection. 30 out of 32 patients had only one reresection of the liver.

## 1. Introduction

The second most common cause of cancer-related deaths worldwide is colorectal cancer (CRC), ranking second in Europe and third in the USA [1]. 

Surgical resection, if possible, is the standard treatment for patients with a localized tumour, but about 50–75% of patients with CRC will develop colorectal liver metastases (CRLM) [2–7]. Even in patients with advanced CRC disease, the liver may be the sole organ with metastases, which is the case in about 30% of the patients [8, 9]. In these cases, a resection of the CRLM may be potentially curative.

Without treatment, patients diagnosed with CRLM have a median survival time around 8–15 months with a 5-year survival rate of 5% [10–13]. With powerful chemotherapy, the median survival time increases up to as much as two years [14].

### 1.1. Objective

The aim was to study the difference between the primary and repeated liver resection for CRLM, especially as comes long-term survival, intraoperative bleeding, and rate of complications.

### 1.2. General Treatment of Colorectal Liver Metastases

Liver resection is, when possible, considered the standard treatment of choice for CRLM. Other types of treatment are methods for local tumour destruction, including radiofrequency ablation. These treatments are most often used in patients with nonresectable liver metastases, but may be used as a part of neoadjuvant and adjuvant treatment, and together with surgical resection in order to improve the results following surgery [15].

The main issue in adjuvant treatment following liver resection concerns chemotherapy. Chemotherapy is otherwise generally palliative in the treatment of metastases of CRC, but may also prolong the survival time [14]. However, chemotherapy can also be used both as neoadjuvant treatment for downsizing the liver metastases, with our without PVE (portal vein embolization) or other local treatment, making otherwise unresectable tumours surgically respectable. Chemotherapy may also be given as adjuvant treatment following liver resection in order to at least lengthen the period until potential tumor recurrence after the initial liver resection for CRLM happens [16, 17].

### 1.3. Resection of Colorectal Liver Metastases

The 5-year survival rate after resection of CRLM varies broadly in different reports, ranging between 15–50%, but in more recent studies the range is usually 40–50% [2, 18–21].

20–30% of patients with CRLM directly fulfil criteria which make them suitable for liver resections. The criteria for operating CRLM have changed over time, from looking on what can be removed, to also include optimization of what will remain of the liver. Overall, with all novel treatment options, an increased number of patients with CRLM can be offered the surgical option [2, 22–24].

### 1.4. Recurrence and Repeat Resection of Colorectal Liver Metastases

Although liver resections are performed with curative intent, 60% will develop recurrent disease. Between 20–30% with recurrence after the first liver resection will have a disease which potentially allows a repeat liver resection. The criteria for surgery are relatively the same as for the initial resection. Survival rates and risk for complications and length of hospital stay are reported similar to that noted after the first resection [15, 25–29].

## 2. Patients and Methods

Medical data on consecutive patients that underwent liver resection due to colorectal adenocarcinoma metastases at the Department of Surgery, Skåne University Hospital, Sweden, during the period 1995–2009, was collected in a database. The information was taken from patients subjected to liver resection for CRLM. Follow-up data was also retrieved for patients who were referred from other hospitals. Four patients registered as having only local intervention actually had a formal liver resection performed and thus entered the database. The database includes a large variety of information, for example, age, intraoperative bleeding, operation time, and complications.

In total, 240 patients had liver resection due to CRLM during the period 1995–2009. Patients who were included in this study had CRLM and underwent a repeated liver resection during the period 1995–2009 at the Department of Surgery, Skåne University Hospital Lund, Sweden. We identified 32 patients, 30 out of which with one reresection of the liver and the other two had two reresections, thus making a total of 34 repeated liver resections. All patients were operated upon with curative intent. The 240 primary liver resections served as control group.

### 2.1. Statistical Methods

The tests used were Mann-Whitney *U* test, Fisher's exact test, Kruskal-Wallis test, and the Kaplan-Meier test.

## 3. Results

There was no 30-day operative mortality or postoperative hospital deaths following the 34 repeat resections. The median age at the primary resection was 66 years compared to 64 at the repeat resection (n.s.). Of the 240 primary resections, 91 were female compared to 14/34 at the repeat resection (38% versus 41%; *P* = 0.710). The primary resection was more often a large (hemihepatectomy or more than 3 segments) resection as compared to the repeat resections (*P* < 0.0001). The median intraoperative blood loss at repeat resection was 1850 mL, significantly higher as compared to the bleeding noted at primary liver resections (in median 1000 mL; *P* = 0.014). This was the fact even though smaller resections had significantly less bleeding compared to large resections (*P* = 0.012). The median hospital stay at a repeat resection was 8 days, that is, the same as at a primary resection (*P* = 0.98). There was no difference in the rate of complications (*P* = 0.568) between primary and repeat liver resections.

There was no difference in the number of patients that received neoadjuvant chemotherapy (within the month prior to the operation) or adjuvant chemotherapy (*P* = 0.826 and *P* = 0.748, resp.).

**Figure 1:**
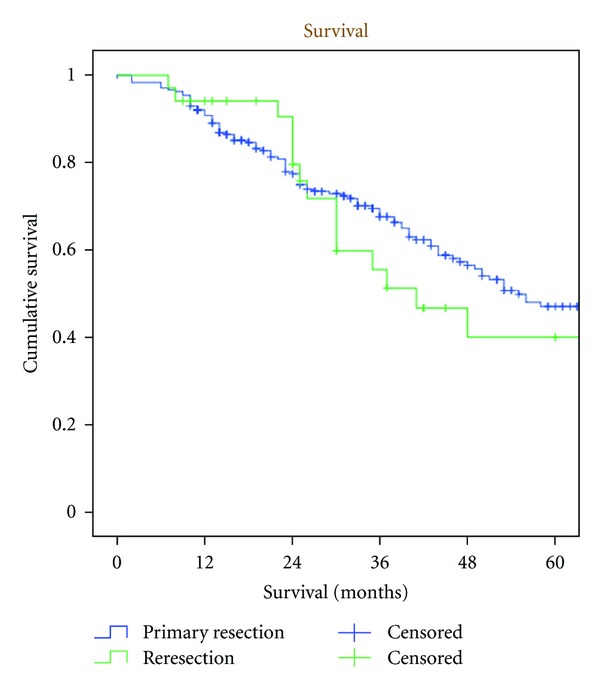
Kaplan-Meier estimate on survival following primary liver resection for colorectal liver metastases and reresection of the liver (n.s.).

There was no difference in survival (Figure 1) between the two groups (*P* = 0.556), despite the fact that repeat resections were less microscopically radical according to the PAD (*P* = 0.046). The tumours were slightly larger at the primary resection, though not significantly (*P* = 0.108).

Increased bleeding at the primary resection (>1000 mL) was associated with a higher risk for both complications (*P* = <0.0001) and an increased length of stay (*P* = 0.004).

A higher ASA-class tended to increase the length of hospital stay (*P* = 0.052). When excluding ASA-class IV from the equation, the difference was not significant (*P* = 0.12). ASA-class did not significantly affect neither bleeding (*P* = 0.092) nor risk for complications (*P* = 0.611; Table 1).

## 4. Discussion

During the last decades, there has been great advancement in the field of management of colorectal liver cancer metastases, including recurrent disease. Repeated resections of the liver for colorectal liver metastases have in most studies proved to be beneficial, even rendering some improvement in cases with multiple metastases. Patients thus tolerated the surgical resection well despite a technically difficult operation on a liver potentially damaged by chemotherapy, and mortality and morbidity do not seem to be higher than that reported following a primary liver resection.

In our study, we noted that the intraoperative bleeding was significantly higher in repeated resections as compared to the primary resection. Previous studies [26] have shown similar results. A probable cause of the increased bleeding may be the altered anatomy, in and around the liver, with scar tissues and adhesions, which thus makes it more difficult to avoid an increase in the intraoperative bleeding. In addition, the repeat resections were more often atypical, suggesting less clear segmental limits, therefore making it an even more challenging operation.

The increased intraoperative bleeding in repeat liver resections did not have a negative effect on outcome considering the rate of complications and length of hospital stay. This was a little surprising, as a bleeding of more than 1000 mL in itself was a risk factor for both an increase in the rate of complications as well as length of stay. An explanation could be the fairly limited number of patients, thereby decreasing the possibilities to draw definite conclusions.

We also found that there were no differences between the two groups in terms of how many that received neoadjuvant or adjuvant chemotherapy. This can be interpreted that patients in both groups are treated equally from an oncological point of view and thereby has the same conditions when subjected to liver surgery. Overall, repeat liver resections are handled in a similar way as primary liver resections. By treating the two groups in the same way one can assume that experience accumulated from primary resections are also valid on repeat resections. This is important, as repeat resections are fairly limited in number, though the overall accumulating data support the safety and benefits of repeat liver resections [5, 30–32].

## 5. Conclusion

Repeated resections of the liver in patients with colorectal liver metastases are associated with an increase in intraoperative bleeding as compared to a primary liver resection. However, this had no effect on the outcome, on survival, rate of complications, or on length of hospital stay. Therefore, repeated liver resections for colorectal liver metastases represent a safe and worthwhile operation with results in similarity with those reported following primary liver resections.

## Figures and Tables

**Table 1:** Primary and repeated liver resection due to colorectal liver metastases—outcome data.

	Primary resection	Repeat resection	*P* value
Age	66 ± 10 years	64 ± 9 years	n.s.
Gender	62% males	59% males	n.s.
Bleeding	1000 ± 1786 mL	1850 ± 1833 mL	0.014
Length of stay	8 ± 8 days	8 ± 3 days	n.s.
Neoadjuvant chemo. administered	33%	31%	n.s.
Adjuvant chemo. administered	39%	36%	n.s.
Clavien grade II+	65%	71%	n.s.
2-year survival	76%	86%	n.s.
